# Y_2_O_3_:Yb^3+^, Tm^3+^/ZnO composite with a heterojunction structure and upconversion function for the photocatalytic degradation of organic dyes

**DOI:** 10.1039/d1ra03066c

**Published:** 2021-07-07

**Authors:** Yuehui Tai, Yu Zhang, Jinlong Sun, Fuyue Liu, Haoran Tian, Qifeng Liu, Caihong Li

**Affiliations:** School of Ecology and Environment, Inner Mongolia University No. 235, University West Road Hohhot China ndlqf@imu.edu.com; School of Chemical Engineering, Inner Mongolia University of Technology No. 45, Aimin Road Hohhot China licaihong@imut.edu.com

## Abstract

Endowing photocatalytic materials with a broader range of light responses is important for improving their performance and solar energy utilization. In this study, a simple sol–gel method was used to prepare Yb^3+^/Tm^3+^-co-doped Y_2_O_3_ upconversion materials and Y_2_O_3_:Yb^3+^, Tm^3+^/ZnO (Y/Z) composite photocatalysts for the photocatalytic degradation of dyes. The Y/Z composite photocatalyst achieved degradation rates of 38%, 95%, and 89% for methyl orange, methylene blue (MB), and acid chrome blue K dye solutions, respectively, within 30 minutes. The degradation efficiency for MB after three cycles of degradation was 86%. The spherical Y_2_O_3_:Yb^3+^, Tm^3+^ particles had diameters of 20–50 nm and attached to the ZnO nanosheets, forming a heterojunction structure with ZnO. Fluorescence spectroscopy showed that Y_2_O_3_:Yb^3+^, Tm^3+^ could convert near-infrared (NIR) light into three sets of ultraviolet light (290, 320, and 360 nm) under NIR excitation. Photoluminescence spectroscopy demonstrated that the photogenerated electron–hole pair recombination probability of the composite photocatalyst was significantly lower than that of ZnO nanosheets, thereby reducing the energy loss during the migration process. Furthermore, the addition of Y_2_O_3_:Yb^3+^, Tm^3+^ to ZnO substantially improved the absorption capacity for ultraviolet light, which enhanced the photocatalytic activity. A possible mechanism for the enhanced photocatalytic performance of the Y/Z composites was proposed based on the synergistic effect of heterojunction formation and the photoconversion process. The composite photocatalyst with upconversion characteristics and heterogeneous structure provides a new strategy for removing organic pollutants from water.

## Abstract 2

Upconversion heterojunction synergistic effect for photocatalytic degradation of organic pollutants.

## Graphical abstract

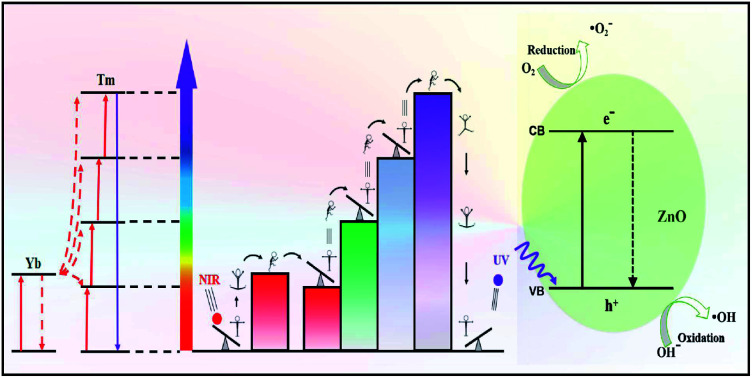

## 1. Introduction

As a green and renewable energy, solar energy has been widely used by humankind and is expected to solve critical problems related to energy shortage and environmental pollution. However, due to the limitation of excitation light source energy, such as photocatalysis, bioapplications, and photodynamic medical technology,^1–6^ it can only use about 50% of the visible light of sunlight, and even 4–5% of ultraviolet (UV) light,^7^ significantly limiting applications and reducing the utilization efficiency of solar energy. Upconverting technology (UPT) can convert two or more low-energy photons in the light source into one high-energy photon,^8–13^ realizing the conversion of low-energy light to high-energy light. Thus, UPT provides new strategies for the efficient use of solar resources to address challenges related to photodynamics in different fields.

In recent years, photocatalytic water splitting using solar energy to produce hydrogen, the mineralization and degradation of organic pollutants, cathodic protection and anticorrosion, and the reduction of high-priced heavy metal ions and CO_2_ have attracted widespread attention.^14–23^ Based on the properties of semiconductors, wide-bandgap photocatalysts often have stronger redox activity. However, these photocatalysts can only be excited by high-energy UV light in the solar spectrum, which is a critical problem that needs to be solved for the practical application of photocatalytic technology. In addition, UV and visible light have a weak ability to penetrate the solution in a heterogeneous photocatalytic system. This causes some photocatalysts to fail to function, thereby limiting the practical application and catalytic efficiency. Therefore, new photocatalysts with near-infrared (NIR) light response and high-efficiency photochemical conversion need to be designed. The use of UPT to modify wide-bandgap photocatalysts allows the upconversion luminescent nanoparticles to convert NIR light into visible and UV light. In this way, NIR light can be used to activate the catalyst and produce photogenerated electron–hole pairs with high redox performance without affecting the high redox potential of the catalyst. In this approach, the high redox activity of the catalyst is retained, and the intense NIR light penetration is taken advantage of to optimize catalyst activity and improve sunlight utilization. Li *et al.*^24^ synthesized a NaYF_4_:Yb, Tm, Er–Pt@MOF/Au composite. Based on the light conversion performance of upconversion nanoparticles (UCNPs), the absorption of the composite was expanded from NIR to UV, thereby improving the hydrogen production performance. Wu *et al.*^25^ co-doped In_2_S_3_ with Yb^3+^ and Tm^3+^ ions to synthesize a composite photocatalyst with a flower-like tetragonal structure. Under full-spectrum simulated sunlight, metallic chromium(vi) was reduced to metallic chromium(iii) within 6 min. The reduction rate reached 99.4%, and the degradation efficiency of rhodium boride (RhB) reached 94.8% within 7 min. The efficiencies were 2.17 and 5.60 times that of single-phase In_2_S_3_, respectively. Liu *et al.*^26^ designed and prepared a CaF_2_:Yb@BiVO_4_ composite photocatalyst that can be excited by NIR light. Modifying the photocatalyst by upconversion materials improved the photocatalytic performance and resulted in excellent photochemical stability, providing a new strategy for the design and application of photocatalytic materials. Lu *et al.*^27^ used a microwave hydrothermal method to assemble CeF_3_ with upconversion performance on g-C_3_N_4_ nanosheets, resulting in a new CeF_3_/g-C_3_N_4_ heterojunction photocatalyst that effectively promotes the separation of photogenerated electrons and holes. Dibenzothiophene was found to have a good desulfurization effect, with the desulfurization rate reaching 84.2% within 3 h. Li *et al.*^28^ prepared Fe^3+^-doped BiOBr:Yb^3+^/Er^3+^ photocatalytic materials that showed good photocatalytic performance for the degradation of RhB dyes under simulated UV, visible, NIR, and full-spectrum light irradiation.

Based on the potential to achieve efficient solar energy utilization and good redox effects by combining UPT and photocatalytic materials,^29–34^ in this study, Y_2_O_3_ was co-doped with Tm^3+^ and Yb^3+^ to form an activator–sensitizer system (Y_2_O_3_:Yb^3+^, Tm^3+^) using a sol–gel method. The effect of the Tm^3+^ and Yb^3+^ doping amount on the photoconversion performance of the UCNPs was then analyzed, and the photoconversion mechanism was evaluated. UCNPs were combined with the wide-bandgap photocatalyst ZnO (3.37 eV) to prepare a Y/Z composite photocatalyst, and its structure, morphology, and photocatalytic activity were studied. Finally, the photocatalytic mechanism of the composite photocatalyst was evaluated.

## 2. Materials and methods

### 2.1 Chemicals and reagents

The reagents used were concentrated nitric acid (HNO_3_), polyethylene glycol (PEG 400), yttrium oxide (Y_2_O_3_), thulium oxide (Tm_2_O_3_), ytterbium oxide (Yb_2_O_3_), sodium citrate (Na_3_C_6_H_5_O_7_·2H_2_O), zinc acetate (Zn(CH_3_COO)_2_·2H_2_O), oxalic acid (C_2_H_2_O_4_·2H_2_O), absolute ethanol (C_2_H_6_O), and deionized water (H_2_O). All reagents were of analytical grade and were used directly without further purification.

### 2.2 Synthesis of Y_2_O_3_:Yb^3+^, Tm^3+^ and Y_2_O_3_:Yb^3+^, Tm^3+^/ZnO

Three hundred mL of a concentrated 68% nitric acid solution and 3 mL of PEG 400 was added and dispersed ultrasonically for 10 min to form a stable dispersion, which was stored in the dark for later use. Y_2_O_3_ (2.5 mmol), a certain amount of Tm_2_O_3_ (0.1%, 0.2%, 0.3%, 0.4%, 0.5%, or 0.6%; mole percentage of Y^3+^) and a certain amount of Yb_2_O_3_ (2%, 6%, 10%, 14%, 18%, 22%, or 26%; mole percentage of Y^3+^) were separately dispersed in 100 mL of the above-prepared concentrated nitric acid solution under ultrasonication for 10 min. The solutions were then stirred at 80 °C until the Y_2_O_3_, Yb_2_O_3_, and Tm_2_O_3_ powders were dissolved and the solutions were clear. Subsequently, the three solutions were mixed, and 1.47 g of sodium citrate metal chelating agent was added followed by ultrasonication for 10 min. After the mixed solution formed a stable precursor solution, it was stirred at constant temperature (80 °C) until the solution became a thick, pale-yellow sol. The sol was sealed in a dark place and aged for 12 h. After aging, the sol was dried at 120 °C for 12 h to obtain a white xerogel precursor. The precursor was calcined in a muffle furnace at 800 °C for 2 h to obtain the Y_2_O_3_:Yb^3+^, Tm^3+^ upconversion luminescent powder.

Ammonium nitrate (0.105 g) was dissolved in 10 mL of zinc acetate solution (0.6 mol L^−1^). A certain amount of Y_2_O_3_:Yb^3+^, Tm^3+^ powder (0.2, 0.4, 0.6, or 0.8 g) was dispersed in 20 mL of oxalic acid in total ethanol solution (1.2 mol L^−1^) under ultrasonic treatment for 10 min to form a stable dispersion. Subsequently, under vigorous stirring, the mixed solution of zinc acetate and ammonium citrate was slowly and uniformly added dropwise to the Y_2_O_3_:Yb^3+^, Tm^3+^ distribution to produce a white sol. After the reaction was completed, the sol was sealed in a dark place and aged for 12 h. The resulting sol was filtered and washed repeatedly with ethanol followed by drying at 80 °C for 6 h to obtain a precursor. The precursor was calcined in a muffle furnace at 500 °C for 3 h to obtain a series of Y_2_O_3_:Yb^3+^, Tm^3+^/ZnO composite materials, abbreviated as (0.2, 0.4, 0.6, 0.8) Y/Z.

### 2.3 Characterization methods

High-resolution transmission electron microscopy (HR-TEM; FEI Tecnai F30, Philips-FEI, Netherlands) and field-emission scanning electron microscopy (SEM; Hitachi S4800, Hitachi Limited, Japan) were used to detect the structural details and morphologies of the as-prepared samples. Elemental composition, crystal structure, and bonding information were determined by energy-dispersive X-ray spectroscopy (EDS; Apollo XT, EDAX Company, US), X-ray diffraction (XRD; Rigaku Ultima IV, Rigaku, Japan), and X-ray photoelectron spectroscopy (XPS; ESCALAB-250Xi, Thermo Fisher Scientific, US), respectively. The fluorescence performance was tested by fluorescence spectroscopy (FLS920, Edinburgh Company, UK), while the light absorption performance was studied by UV-visible (UV-Vis) diffuse reflectance spectrophotometry (UV-1800, Shimadzu Company, Japan). All fluorescence tests used a 980 nm NIR laser as the excitation light source (LWIRL980, Laserwave Beijing, China).

### 2.4 Photocatalytic experiments

**Fig. 1:**
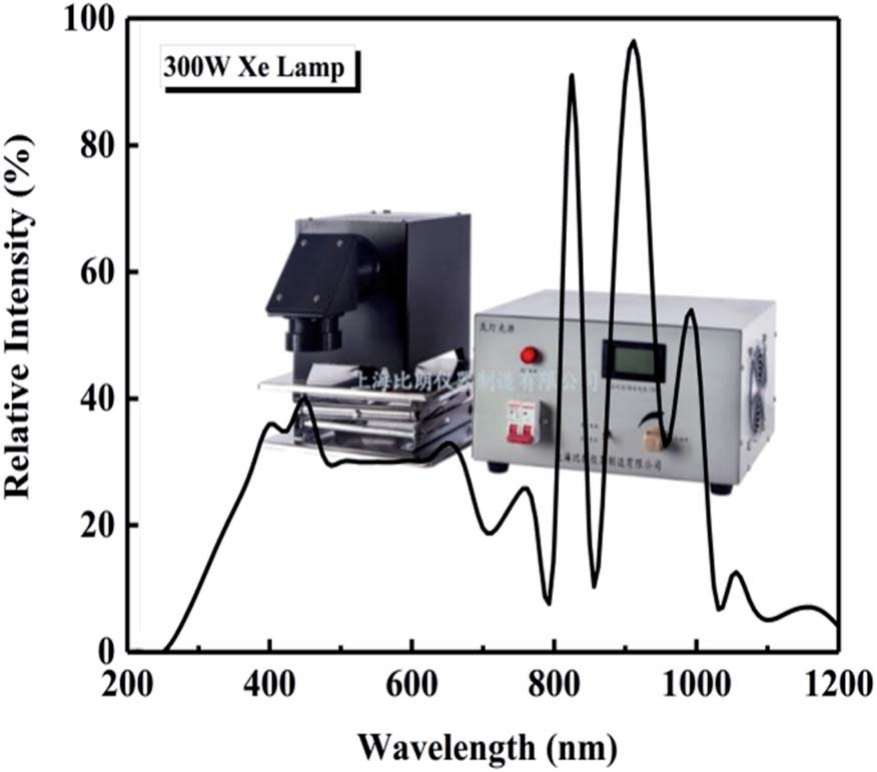
Intensity spectrum of the Xe lamp.

The photocatalytic degradation of three organic dyes, methylene blue (MB), methyl orange (MO), and acid chrome blue K (ACBK), was studied at a dye concentration of 20 mg L^−1^. The volume of the dye solution was 100 mL, and the amount of photocatalyst was 50 mg. Before starting the experiment, the solution to be degraded and the photocatalyst were mixed uniformly and placed in a dark photocatalytic reactor (BL-GHX-V, Shanghai Birang, China) followed by stirring for 30 min to reach adsorption equilibrium. A 300 W Xe lamp (GHX-Xe-300, Shanghai Birang, China) was used for irradiation; the intensity spectrum of the Xe lamp is shown in Fig. 1. The distance between the light source and the liquid surface was 10 cm. The temperature of the dye solution was kept at 25 °C throughout the degradation process using a condensed water system.

## 3. Results and discussion

### 3.1 XRD

**Fig. 2:**
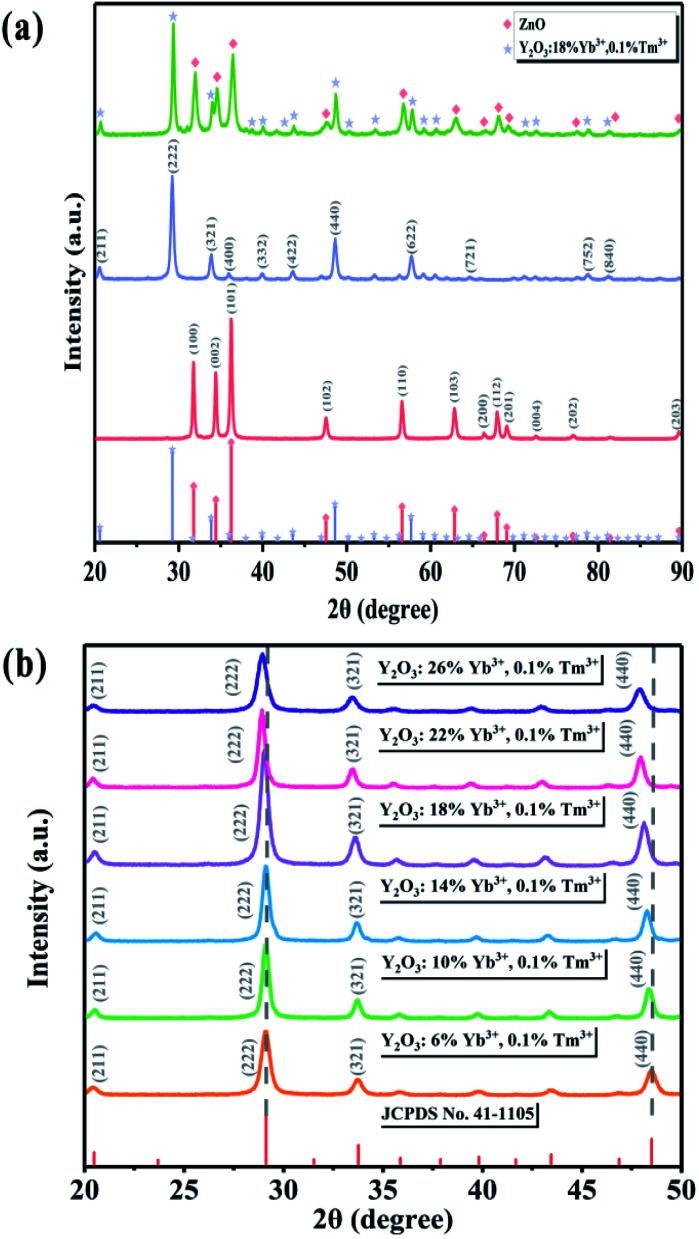
(a) XRD spectra of ZnO, UCNPs, and Y/Z. (b) XRD patterns of UCNPs containing different amounts of Yb ions.

The XRD patterns of ZnO along with the UCNPs and Y/Z composites are shown in Fig. 2a. All three materials had high crystallinity. ZnO (JCPDS No. 99-0111) had a hexagonal wurtzite crystal structure, while the UCNPs had a Y_2_O_3_ (JCPDS No. 41-1105) cubic crystal structure. Fig. 2a shows that the diffraction peak position did not change after ZnO was combined with the UCNPs, indicating that the UCNPs did not enter the ZnO crystal structure. Further, no other impurity peaks appeared, indicating that a relatively pure Y/Z composite was prepared. The microscale doping of Tm^3+^ and Yb^3+^ caused the diffraction peak of Y_2_O_3_ to shift slightly to the left, as shown in Fig. 2b. Because they have the same valence and similar sizes, Tm^3+^ and Yb^3+^ can enter the Y_2_O_3_ lattice and replace Y^3+^. According to the Bragg equation,^35^ the distance between the crystal planes is inversely proportional to the diffraction angle; thus, doping with Tm^3+^ and Yb^3+^ altered the parameters of the Y_2_O_3_ unit cell and increased the crystal plane spacing, resulting in an overall shift of the diffraction peak to the left.

### 3.2 Morphology and microstructural characterization

**Fig. 3:**
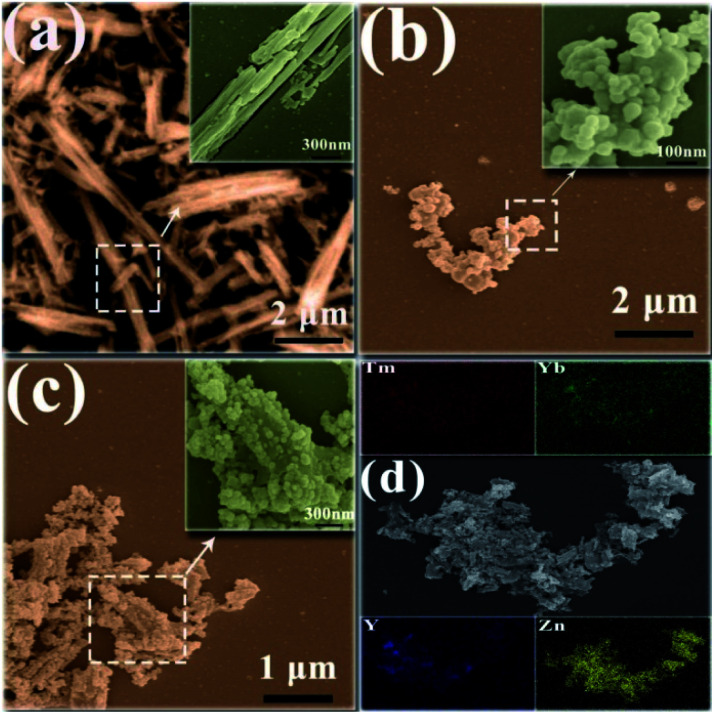
SEM images of (a) pristine ZnO, (b) pristine UCNPs, and (c) the Y/Z nanocomposite. (d) EDS images of the Y/Z nanocomposite.

The high-resolution and low-resolution SEM images and EDS spectra of ZnO, the UCNPs, and the Y/Z composites are shown in Fig. 3. ZnO formed long flakes, and the high-magnification image (Fig. 3a) shows gaps between the ZnO flakes. This structure results in a large specific surface area of 10.45 m^2^ g^−1^ and an adsorption average pore diameter of 6.92 nm, as shown in Table 1. This high specific surface area would facilitate pollutant adsorption.^36^ A nanosheet structure provides a large number of loading sites for UCNPs.^37^ The SEM images of the UCNPs show that the particles were spherical at the nanometer scale, with particle sizes ranging from 30 to 100 nm (Fig. 3b). The SEM image of the composite material (Fig. 3c) shows tiny UCNPs dispersed on the surfaces of the ZnO flakes. This indicates that after high-temperature treatment, the UCNPs formed nanoparticles with higher crystallinity on the surfaces of the ZnO nanosheets, producing a heterogeneous composite structure. The EDS spectrum of the Y/Z composite material shows that Y and Zn were uniformly distributed in the sample particles (Fig. 3d). Trace amounts of doped Yb and Tm were also observed.

**Table 1:** ZnO specific surface area

Sample	BET surface area (m^2^ g^−1^)	Adsorption average pore diameter (nm)
ZnO	10.45	6.92

**Fig. 4:**
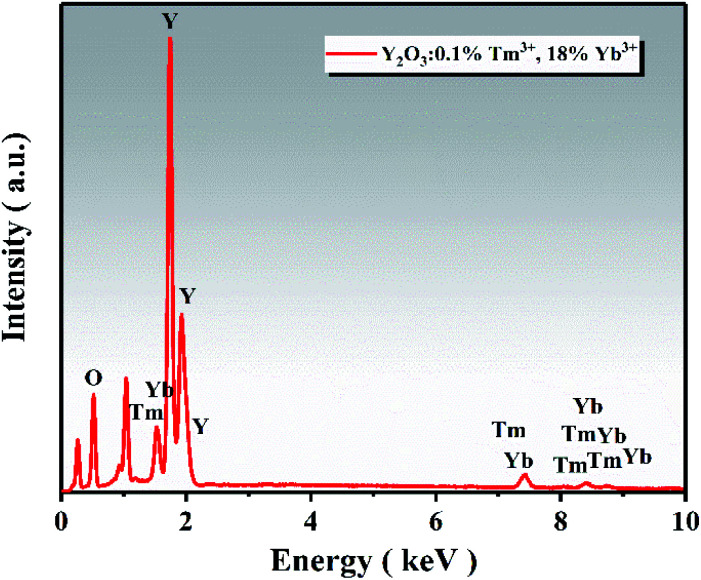
EDS spectrum of the UCNPs.

**Fig. 5:**
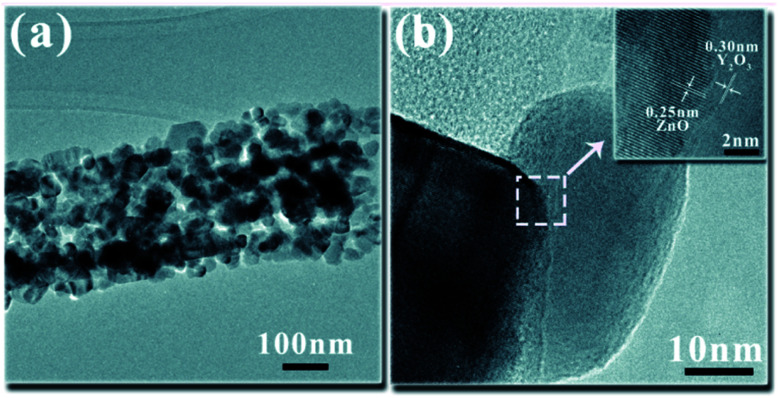
HRTEM images of the Y/Z nanocomposite. The inset shows the interplanar spacing.

The EDS spectrum of the UCNPs is presented in Fig. 4, showing that the UCNPs were composed of O, Y, Yb, and Tm elements. The atomic percentage of each element is shown in Table 2. TEM was employed to further verify the existence of UCNPs on the surface of ZnO in the Y/Z composites. High-resolution TEM revealed many lattice fringes with inconsistent orientations at the interface between the UCNPs and ZnO in the Y/Z composites (Fig. 5). The contact surface between the UCNPs and ZnO had a polycrystalline structure with lattice spacings of 0.25, 0.26, and 0.30 nm, consistent with the (002) and (101) crystal planes of hexagonal ZnO and the (222) crystal plane of Y_2_O_3_, respectively.^38,39^ This indicates that the UCNPs and ZnO were in close contact, forming a heterojunction. A potential difference will be generated between the two sides of this heterostructure, facilitating the separation of photogenerated electron–hole pairs and improving the separation efficiency, thereby enhancing the catalytic activity.^40–45^

**Table 2:** Atomic and weight percentages of each element

Element	Atomic percentage (%)	Weight percentage (%)
O	77.84	35.62
Y	18.81	47.83
Tm	0.21	1.01
Yb	3.14	15.53

### 3.3 Surface structure analysis

**Fig. 6:**
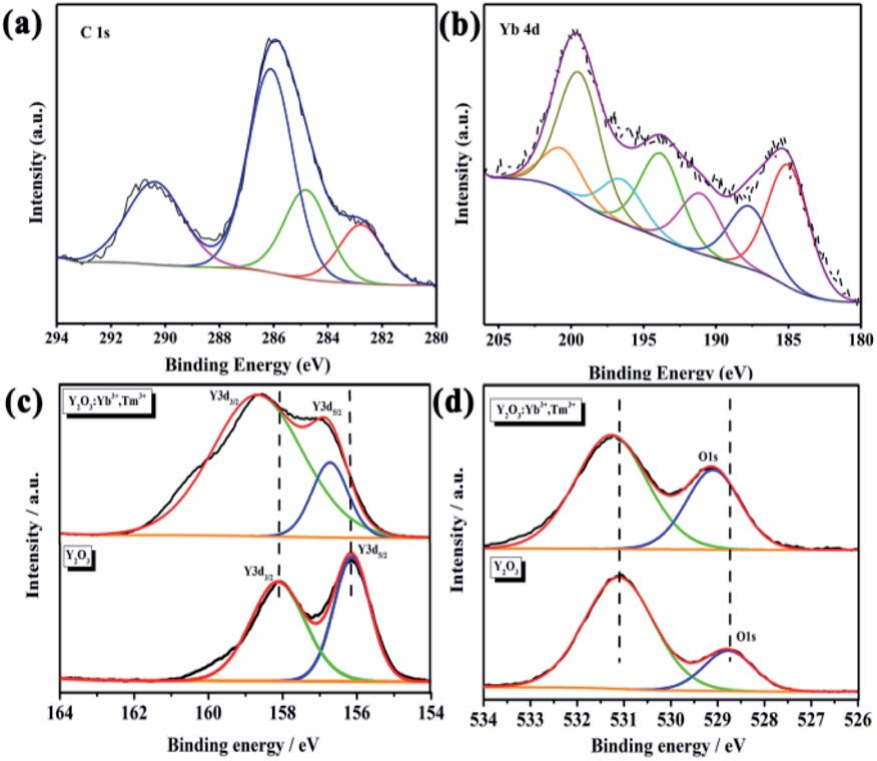
(a) C1s, (b) Yb4d, (c) Y3d, and (d) O1s XPS spectra of the UCNPs.

XPS was used to further characterize the elements contained in the UCNPs and their valences. All peaks were calibrated using carbon C–C/C–H binding energy of 284.8 eV in the C1s adsorption spectrum. The C1s spectrum is shown in Fig. 6a. Fig. 6b shows the Yb4d scan, which demonstrates the existence of Yb in the UCNPs. However, XPS is a surface analysis technique; thus, XPS failed to detect Tm in the Tm4d scan due to the trace doming amount of Tm^3+^. In the Y3d spectrum of pure Y_2_O_3_, the binding energies of 158.08 and 156.13 eV correspond to Y3d_3/2_ and Y3d_5/2_,^46^ respectively, indicating that Y had a +3 valence and existed in the cubic Y_2_O_3_ phase (Fig. 6c). In the spectrum of the UCNPs, the Y3d binding energies are shifted to 158.73 and 156.88 eV because Tm^3+^ and Yb^3+^ entered the host Y_2_O_3_ lattice by isomorphic replacement. Thus, Tm^3+^ and Yb^3+^ replaced Y in the lattice, which affected the number of Y–O bonds and caused a shift in the binding energy. The O1s peak of Y_2_O_3_ in Fig. 6d is relatively broad and asymmetric, indicating at least two combined oxygen states. The XPS peak at 528.8 eV is attributed to lattice oxygen, while that at 531.3 eV is attributed to surface-adsorbed oxygen. These two binding energies represent the two chemical forms of oxygen.^46,47^ In the O1s spectrum of the UCNPs, the position and intensity of the signal corresponding to surface-adsorbed oxygen have not changed. However, the binding energy of lattice oxygen has shifted, and the percentage of total oxygen has increased. Thus, some adsorbed oxygen was changed to lattice oxygen. Tm^3+^ and Yb^3+^ replaced Y^3+^ in the lattice, resulting in lattice distortion and the accumulation of strain energy. Combined with the deviation in the XRD peak position of the UCNP powder, these results indicate that Tm^3+^ and Yb^3+^ entered the Y_2_O_3_ lattice and replaced Y^3+^. In addition, no Tm or Yb oxide phases were found in the XRD pattern, indicating that doping with rare earth ions at low concentration does not generate Tm_2_O_3_ and Yb_2_O_3_ phases.

### 3.4 Luminous performance

**Fig. 7:**
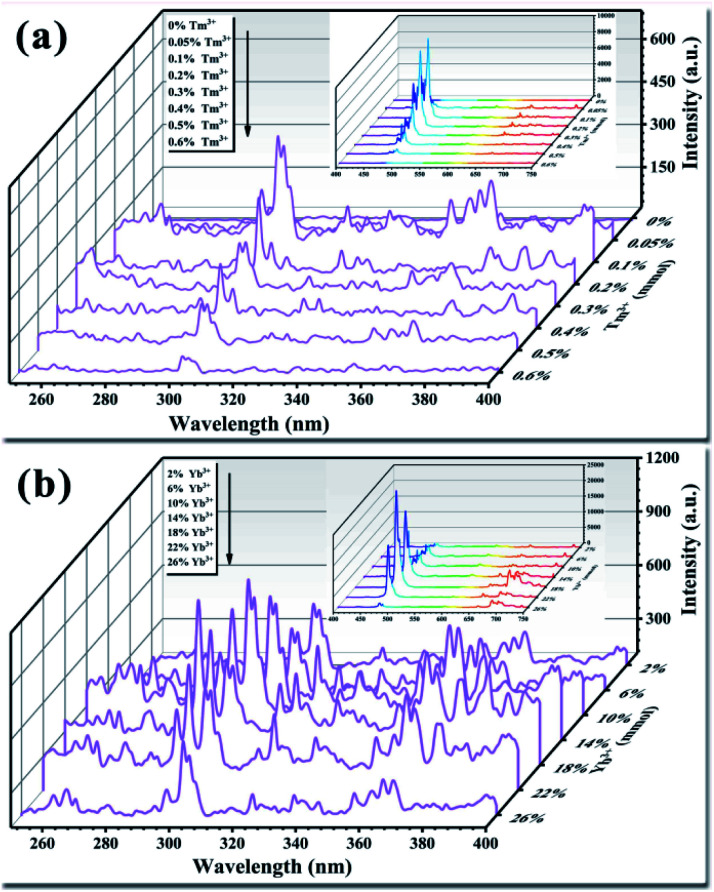
(a) Effect of the activator Tm^3+^ on the luminescence properties of UCNPs. (b) Effect of the sensitizer Yb^3+^ on the luminescence properties of UCNPs. The inset shows the luminous performance in the visible light range (400–750 nm).

The light conversion performance of UCNPs doped only with Tm^3+^ under 980 nm NIR irradiation is shown in Fig. 7a. Doping with trace amounts of Tm^3+^ effectively improved the luminescence performance of the UCNPs. Among the Tm^3+^ doping concentrations, 0.1% (mole percentage of Y^3+^) produced the highest luminous intensity in the UV and visible bands. As the Tm^3+^ doping amount increased, fluorescence quenching occurred, resulting in a significant reduction in the fluorescence intensity of the entire band. Finally, when the doping amount was increased to 0.6%, the fluorescence emission peak disappeared. Tm^3+^ is the activator in the upconversion material and acts as the luminescent center.^48^ Multiple energy levels are distributed inside Tm^3+^, and the positions of the energy levels meet the needs for UV light conversion. Trace doping can increase the fluorescence intensity by an order of magnitude. As the amount of Tm^3+^ doped in the crystal lattice gradually increases, the distance between each Tm^3+^ emission center in the crystal lattice decreases. This phenomenon increases the rate of energy transfer between Tm^3+^ ions, resulting in an intense energy exchange until the energy is transferred to the quenching center. At this point, fluorescence quenching occurs, resulting in a decrease in fluorescence intensity.^49^ Studies have found that UCNPs doped with activators are prone to fluorescence quenching.^50^ The results of the present study show that the optimal amount of activator Tm^3+^ in the UCNPs was 0.1%. The upconversion luminescence of the UCNPs after doping with Tm^3+^ demonstrates that trace amounts of Tm^3+^ were successfully doped into the Y_2_O_3_ crystal lattice.

The light conversion performances of UCNPs co-doped with Tm^3+^ (0.1%) and Yb^3+^ at different doping concentrations are shown in Fig. 7b. When the Yb^3+^ doping amount was 18% (mole percentage of Y^3+^), the light conversion performance of the UCNPs was 3.5 times higher than that achieved by doping with Tm^3+^ only, while the fluorescence peak position was not changed. As the amount of doped Yb^3+^ increased, fluorescence quenching occurred, and the fluorescence intensity was significantly reduced. Yb^3+^ has only one excited-state energy level, and the cross-sectional absorption area of the transition between energy levels is relatively large. Moreover, Yb^3+^ easily resonates with other lanthanide ions. Yb^3+^ can absorb energy from the light source and transfer it to Tm^3+^; thus, the light conversion ability varies with the amount of doped Yb^3+^.^51–53^ As the amount of Yb^3+^ increases, the energy transferred to Tm^3+^ gradually increases, prompting Tm^3+^ to transition to a higher energy level. The doping amount of Yb^3+^ also plays a role in regulating the ion spacing in the lattice. As the amount of Yb^3+^ doping increases, the distance between Tm^3+^ and Yb^3+^ in the crystal lattice decreases, and the probability of energy transfer between ions increases. This reduces the likelihood of cross-relaxation between ions, reduces energy loss, and improves the light conversion ability of the UCNPs.^54–56^ However, when the amount of doped Yb^3+^ is too high, Yb^3+^ and Tm^3+^ in the crystal lattice become close to each other and interact strongly with each other, resulting in a short lifetime of Yb^3+^ ions at the excited-state energy level. In addition, too much Yb^3+^ will generate ion pairs or clusters, decreasing the number of ions that play a sensitizing role and reducing the light conversion ability.^57^

### 3.5 UV-Vis diffuse reflectance spectra

**Fig. 8:**
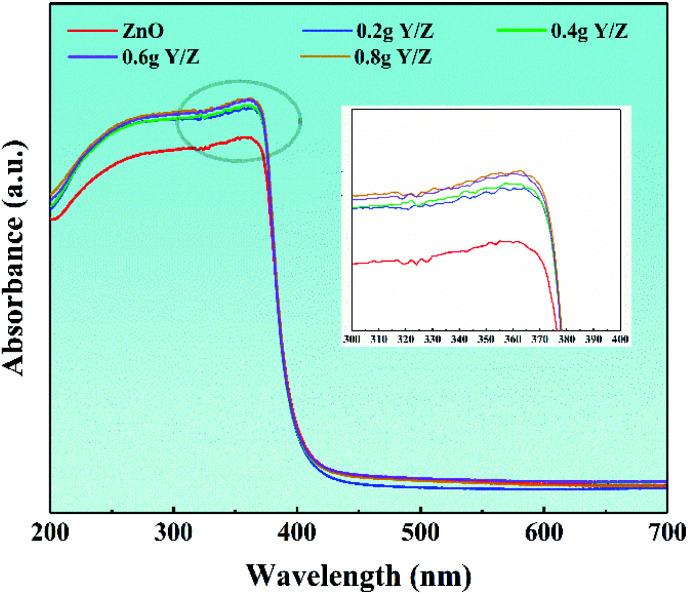
UV-Vis spectra of ZnO and the Y/Z composites. The inset shows the local amplification.

Fig. 8 shows the ultraviolet-visible diffuse reflectance spectra of ZnO nanosheets and a series of Y/Z composite materials. In the ultraviolet region of the spectrum, the absorption thresholds of all materials are concentrated around 380 nm, which corresponds to the characteristic absorption threshold of ZnO forbidden bandwidth (3.37 eV).^58,59^ Simultaneously, in the ultraviolet absorption range, the light absorption intensity of the series of Y/Z composite materials is significantly higher than that of ZnO nanoparticles, and as the amount of UCNPs powder added increases, the light absorption intensity increases accordingly. This increase in light absorption capacity contributes to improving the composite material's photocatalytic performance.^60,61^

### 3.6 Photoluminescence (PL) spectroscopy

**Fig. 9:**
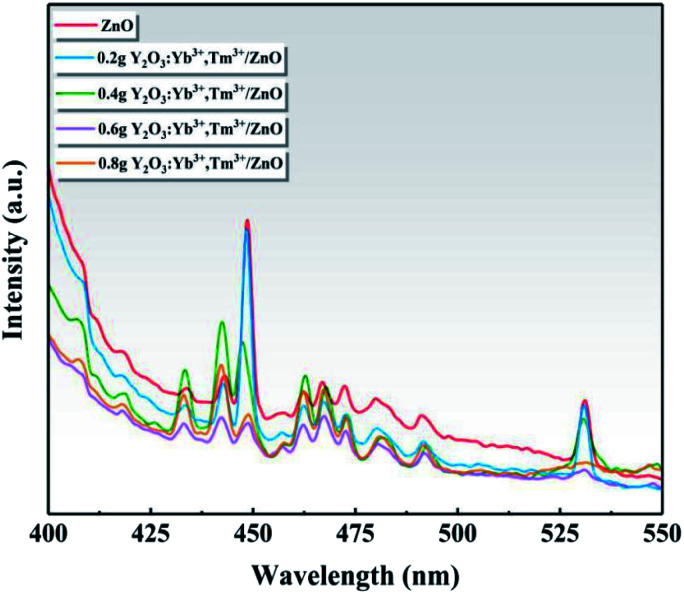
Photoluminescence spectra of ZnO and the Y/Z composites.

Fig. 9 shows the PL spectra of ZnO and the series of Y/Z composite materials at a 360 nm excitation wavelength. A strong PL luminescence signal was observed in the range of 400–550 nm, and prominent fluorescence luminescence peaks appeared at 448 and 530 nm. Compared to ZnO, the PL intensity of the Y/Z composite material was significantly reduced, and the fluorescence peak was quenched to a large extent upon the addition of UCNPs. Among the Y/Z composites, 0.6 Y/Z had the lowest fluorescence peak intensity at 448 and 530 nm. This indicates that the energy lost in the form of electron–hole pair recombination was lowest in 0.6 Y/Z, which is beneficial for photocatalytic activity. The UCNPs and ZnO nanosheets formed a heterojunction structure that effectively inhibited photogenerated electron–hole recombination, improved the separation efficiency of photogenerated electron–hole pairs, and facilitated electron transfer.

### 3.7 Photocatalytic activity

**Fig. 10:**
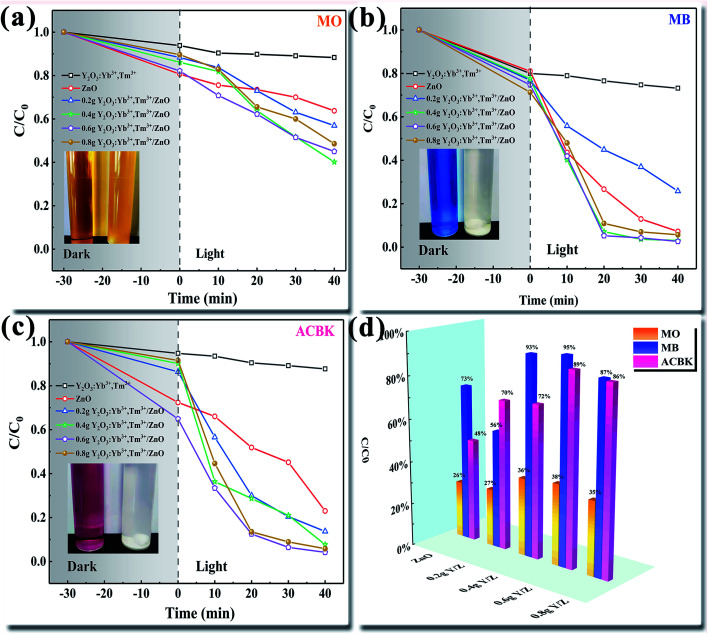
Degradation performances of the prepared photocatalysts for different dyes (a) MO (b) MB (c) ACBK and (d) degradation efficiency within 30 min.

The degradation efficiencies of the ZnO nanosheets, UCNPs, and Y/Z composite materials for the photocatalytic degradation of different dyes (MO, MB, and ACBK) were evaluated through photocatalytic activity tests under irradiation by a 300 W Xe lamp (Fig. 10). Before irradiation, the degradation system was stirred in a dark photocatalytic reactor for 30 min to reach adsorption equilibrium. As shown in Fig. 10a, the degradation rate of MO dye by pure ZnO was only 36% within 40 min. The addition of UCNPs significantly improved the photocatalytic performance. The degradation efficiency of the composite photocatalyst for MO dye was 60% within 40 min, 24% higher than that of the ZnO nanosheets. Fig. 10b shows that the ZnO nanosheets had a good degradation efficiency for MB dye (93% in 40 min). After the addition of UCNPs, the 0.6 Y/Z composite photocatalyst achieved a degradation efficiency of 97% for MB dye within 20 min. Fig. 10c shows that the degradation rate of ACBK dye by pure ZnO was 77% within 40 min. Upon the addition of UCNPs, the photocatalytic performance of ZnO first increased and then decreased. Among the composite photocatalysts, 0.6 Y/Z had the highest photocatalytic activity, with the degradation efficiency for ACBK dye reaching 96% within 20 min. To facilitate the comparison of material properties, the degradation efficiency within 30 min was selected for analysis. Irradiation by the 300 W Xe lamp significantly improved the photocatalytic performances of the Y/Z composite photocatalysts for the three tested dyes (Fig. 10d). Among the composite photocatalysts, 0.6 Y/Z had the best photocatalytic performance, with degradation efficiencies of 38%, 95%, and 89% for MO, MB, and ACBK, respectively. According to the previous characterization results, the activity of the 0.6 Y/Z composite photocatalyst can be attributed to the photoconversion ability of the doped UCNPs and the heterojunction structure, which inhibits the recombination of photogenerated electron–hole pairs to improve the photocatalytic efficiency. The improvement in photocatalytic activity was consistent with the results of PL spectroscopy.

**Fig. 11:**
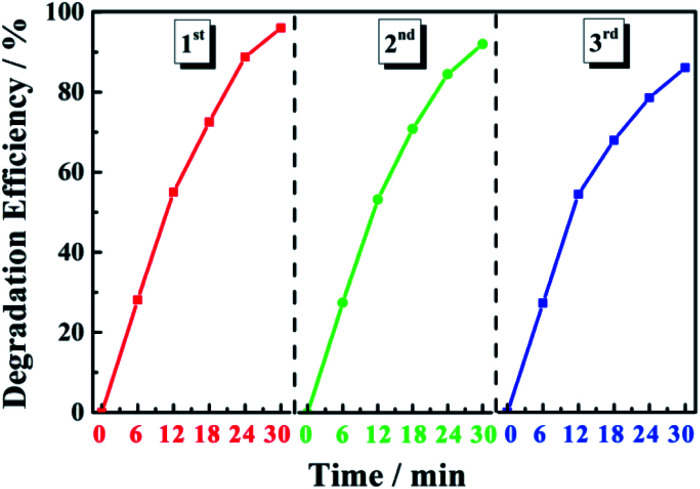
Degradation efficiency of the 0.6 Y/Z photocatalyst during three cycles of MB degradation.

Photocatalyst stability was evaluated through cyclic degradation experiments using MB dye (20 mg L^−1^) as the simulated pollutant. In the first three cycles of degradation, the degradation efficiency of the 0.6 Y/Z composite photocatalyst was 96%, 92%, and 86% within 30 min, respectively (Fig. 11). These results indicate that the catalyst had good recycling performance and good stability due to the strong bonding between the UCNPs and ZnO nanosheets.

### 3.8 Mechanistic research

**Fig. 12:**
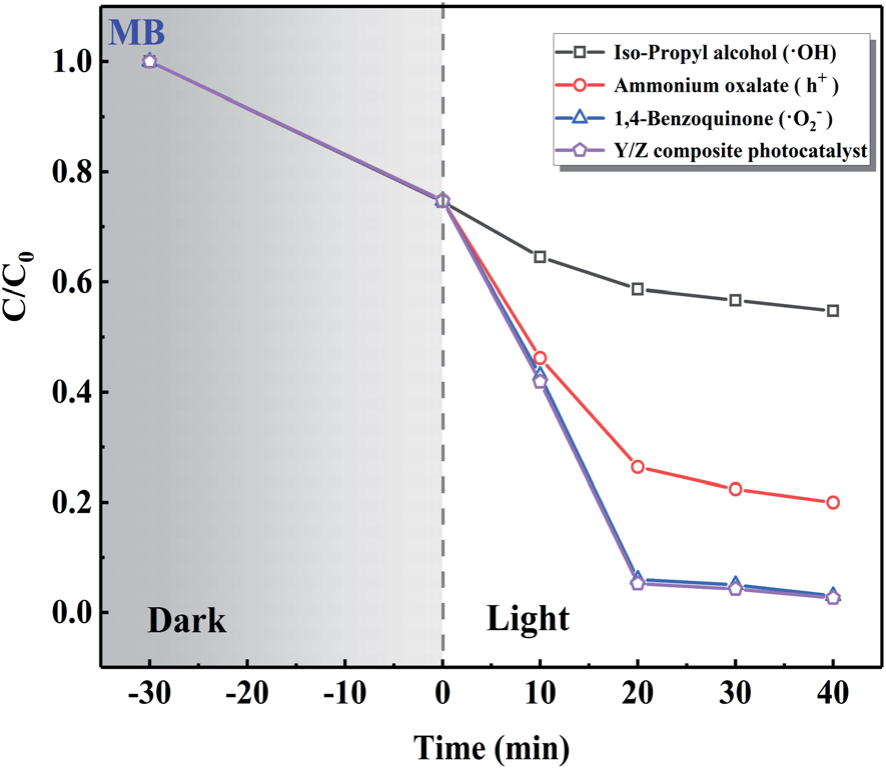
Results of free radical capture experiments.

The results of radical trapping experiments using the Y/Z composite photocatalyst are shown in Fig. 12. The 0.6 Y/Z composite photocatalyst (50 mg) was ultrasonically dispersed in 100 mL of MB dye solution followed by the addition of ˙OH trapping agent isopropyl alcohol, h^+^ trapping agent ammonium oxalate, or ˙O^2−^ trapping agent 1,4-benzoquinone. After irradiation by a Xe lamp for 40 min, the solution containing isopropyl alcohol was significantly affected. This demonstrates that ˙OH radical is the main active species in the Y/Z composite photocatalyst.

**Fig. 13:**
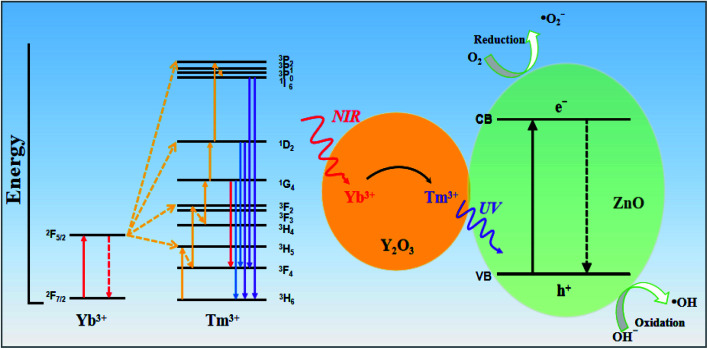
Schematic showing the photocatalytic mechanism of the Y/Z composite photocatalyst.

Based on the above results, Fig. 13 shows a proposed photocatalytic mechanism for the Y/Z composite catalyst. The photocatalytic effect of the Y/Z composite photocatalyst depends on energy transfer between the UCNPs and ZnO nanosheets and the formation of a heterojunction structure between the UCNPs and ZnO nanosheets. First, the UCNPs convert NIR light into visible and UV light. Under irradiation by 980 nm NIR light from a Xe lamp, the ^2^F_7/2_ energy level of ground-state Yb^3+^ absorbs NIR light, causing Yb^3+^ to migrate to the ^2^F_5/2_ energy level and transfer energy to Tm^3+^. Tm^3+^ is excited from the ^3^H_6_ energy level to the ^3^H_5_ energy level followed by a nonradiative relaxation transfer to the ^3^F_4_ level. After reaching the ^3^F_4_ level, Tm^3+^ absorbs the energy transferred from Yb^3+^ and transitions to the ^3^F_2_ and ^3^F_3_ levels followed by rapid nonradiative relaxation to the ^3^F_4_ level. After two-photon energy absorption, Tm^3+^ receives the energy from Yb^3+^ and transitions to the ^1^G_4_ energy level. Since the upconversion process has not yet ended, Tm^3+^ continues to transition to the ^1^D_2_ energy level. Finally, Tm^3+^ in the ^1^D_2_ energy level absorbs energy again. Part of Tm^3+^ transitions to the ^3^P_2_ level and undergoes nonradiative relaxation to the ^1^I_6_ level, and the function returns to the ^3^H_6_ level and emits 360 nm UV light. Tm^3+^ at the ^1^I_6_ level returns to the low-energy ^3^F_4_ and ^3^H_6_ levels and emits 320 and 290 nm UV light. After accumulating multiple NIR photons in the upconversion process, the UCNPs will convert three sets of higher-energy UV light for use by the catalyst. Subsequently, the ZnO nanosheets are excited by the UV light converted by the UCNPs and the UV light from the Xe lamp, resulting in the generation of highly oxidizing ˙OH active groups for photocatalysis. In addition, the combination of UCNPs and nanoscale ZnO can enhance the absorption capacity of UV light and form a composite heterojunction structure. The formation of this structure accelerates photogenerated electron–hole separation and reduces the probability of photogenerated electron–hole recombination. The synergy between the upconversion process and the heterojunction structure explains the improvement in the photocatalytic activity of the ZnO nanosheets upon UCNP addition.

## 4. Conclusions

In summary, a Y_2_O_3_:Yb^3+^, Tm^3+^/ZnO composite heterojunction photocatalyst with upconversion function was successfully synthesized using a direct and straightforward sol–gel method. The morphology, composition, and performance of the photocatalyst were characterized. Under irradiation by an Xe lamp, the catalyst showed good photocatalytic activity and stability for the degradation of three dyes (MB, MO, and ACBK). The photocatalytic activity of ZnO was greatly enhanced by combining the ZnO nanosheets with UCNPs. This improvement in photocatalytic activity was attributed to the synergistic effect of heterojunction formation and UV light conversion by the UCNPs. Moreover, the combination of UCNPs and ZnO extended the photoresponse range of the catalyst to the NIR region and improved the sunlight absorption and utilization rates. The formed heterojunction structure enabled the effective separation of photogenerated electron–hole pairs, inhibited carrier recombination, and improved the photocatalytic activity of the composite catalyst. By demonstrating that the addition of upconversion materials can endow photocatalysts with new capabilities and form heterojunction structures, this research provides unique solutions to the problems of insufficient visible-light penetration, low solar energy utilization, and easy recombination of electrons and holes in heterogeneous photocatalytic systems.

## Conflicts of interest

All authors contributed to the discussion. The authors declare no competing financial interest.

## Supplementary Material

## References

[cit1] Wang W., Tade M. O., Shao Z. (2018). Prog. Mater. Sci..

[cit2] Zhang X., Zhang X., Feng K., Hu X., Fan J., Liu E. (2021). RSC Adv..

[cit3] Yang M., Huang J., Fan J., Du J., Pu K., Peng X. (2020). Chem. Soc. Rev..

[cit4] Luo X., Xue B., Feng G., Zhang J., Lin B., Zeng P. (2019). J. Am. Chem. Soc..

[cit5] Li X., Lee S., Yoon J. (2018). Chem. Soc. Rev..

[cit6] Lv W., Zhang Z., Zhang K., Yang H., Liu S., Xu A. (2016). Angew. Chem., Int. Ed..

[cit7] Fan W., Bai H., Shi W. (2014). CrystEngComm.

[cit8] Duan C., Liang L., Li L., Zhang R., Xu Z. (2018). J. Mater. Chem. B.

[cit9] Zhou J., Liu Q., Feng W., Sun Y., Li F. (2015). Chem. Rev..

[cit10] Luo Z., Zhang L., Zeng R., Su L., Tang D. (2018). Anal. Chem..

[cit11] Zhu X., Li J., Qiu X., Liu Y., Feng W., Li F. (2018). Nat. Commun..

[cit12] Liu C., Liu B., Zhao J., Di Z., Chen D., Gu Z. (2020). Angew. Chem., Int. Ed..

[cit13] Liu H., Yan X., Shen L., Tang Z., Liu S., Li X. (2019). Mater. Horiz..

[cit14] Guo S., Li X., Li J., Wei B. (2021). Nat. Commun..

[cit15] Cao S., Wu X., Chen Y., Qiu S., Liu X., Sun C. (2020). Nano Today.

[cit16] Cao S., Piao L. (2020). Angew. Chem., Int. Ed..

[cit17] Wu J., Wang W., Tian Y., Song C., Qiu H., Xue H. (2020). Nano Energy.

[cit18] Li S., Chen J., Hu S., Wang H., Jiang W., Chen X. (2020). Chem. Eng. J..

[cit19] Hossain S. M., Park H., Kang H., Mun J., Tijing L., Rhee I. (2021). Chemosphere.

[cit20] Liu X., Jing X., Zhao Y., Wang W., Yu L., Sun M. (2020). ACS Appl. Electron. Mater..

[cit21] Kuriki R., Sekizawa K., Ishitani O., Maeda K. (2015). Angew. Chem., Int. Ed..

[cit22] Won D. I., Lee J. S., Ji J. M., Jung W. J., Son H. J., Pac C. (2015). J. Am. Chem. Soc..

[cit23] Das S., Perez-Ramirez J., Gong J., Dewangan N., Hidajat K., Gates B. C. (2020). Chem. Soc. Rev..

[cit24] Li D., Yu S., Jiang H. (2018). Adv. Mater..

[cit25] Wu Z., Yuan X., Zeng G., Jiang L., Zhong H., Xie Y. (2018). Appl. Catal., B.

[cit26] Liu X., Di W., Qin W. (2017). Appl. Catal., B.

[cit27] Lu X., Chen F., Qian J., Fu M., Jiang Q., Zhang Q. (2020). J. Rare Earths.

[cit28] Li Y., Yao L., Xu D., Hu Y., Yang S., Zhang Y. (2019). Inorg. Chem. Front..

[cit29] Zhang C., Zhang C., Zhang Z., He T., Mi X., Kong T. (2020). Opto-Electron. Adv..

[cit30] Liang T., Wang Q., Li Z., Wang P., Wu J., Zuo M. (2020). Adv. Funct. Mater..

[cit31] Tou M., Luo Z., Bai S., Liu F., Chai Q., Li S. (2017). Mater. Sci. Eng., C.

[cit32] Xu Z., Quintanilla M., Vetrone F., Govorov A. O., Chaker M., Ma D. (2015). Adv. Funct. Mater..

[cit33] Ravetz B. D., Pun A. B., Churchill E. M., Congreve D. N., Rovis T., Campos L. M. (2019). Nature.

[cit34] Liu X., Qiu J., Xu X., Zhou D. (2016). J. Nanosci. Nanotechnol..

[cit35] Sun Q., Zhou H., Zhu H., Qi H., Hu L., Yue Z. (2016). J. Mater. Sci.: Mater. Electron..

[cit36] Mekasuwandumrong O., Pawinrat P., Praserthdam P., Panpranot J. (2010). Chem. Eng. J..

[cit37] Zhou S., Ma D., Cai P., Chen W., Huang S. (2014). Mater. Res. Bull..

[cit38] Li T., Li B., Ji Y., Wang L. (2018). Polymers.

[cit39] Borschel C., Ronning C., Hofsäss H., Giussani A., Zaumseil P., Wenger C. (2009). J. Vac. Sci. Technol., B: Microelectron. Nanometer Struct.--Process., Meas., Phenom..

[cit40] Ma D., Wang Z., Shi J., Zou Y., Lv Y., Ji X. (2020). J. Mater. Chem. A.

[cit41] Xu Q., Zhang L., Cheng B., Fan J., Yu J. (2020). Chem.

[cit42] Low J., Yu J., Jaroniec M., Wageh S., Al-Ghamdi A. A. (2017). Adv. Mater..

[cit43] Wang H., Zhang L., Chen Z., Hu J., Li S., Wang Z. (2014). Chem. Soc. Rev..

[cit44] Feng H., Liang L., Liu Y., Huang Z., Li L. (2019). Appl. Catal., B.

[cit45] Zheng Y., Fan M., Li K., Zhang R., Li X., Zhang L. (2020). Catal. Sci. Technol..

[cit46] Barreca D., Battiston G. A., Berto D., Gerbasi R., Tondello E. (2001). Surf. Sci. Spectra.

[cit47] Moulder J. F., Chastain J., King R. C. (1992). Chem. Phys. Lett..

[cit48] Li H., Hao H., Jin S., Guo W., Hu X., Hou H. (2018). Adv. Powder Technol..

[cit49] Richardson R. R., Irelan P. T., Howey D. A. (2014). J. Power Sources.

[cit50] Wang F., Liu X. (2008). J. Am. Chem. Soc..

[cit51] Lojpur V., Nikolic M., Mancic L., Milosevic O., Dramicanin M. D. (2013). Ceram. Int..

[cit52] Kodama T., Fujii M., Nakano T., Imakita K., Hayashi S. (2013). Opt. Mater..

[cit53] Liang L., Wu H., Hu H., Wu M., Su Q. (2004). J. Alloys Compd..

[cit54] Agrawal S., Dubey V. (2019). J. Radiat. Res. Appl. Sci..

[cit55] Wild J., Meijerink A., Rath J. K., Sark W., Schropp R. (2011). Energy Environ. Sci..

[cit56] Yao N., Huang J., Fu K., Deng X., Ding M., Shao M. (2015). Electrochim. Acta.

[cit57] Li Y., Guo J., Liu X., Aidilibike T., Qin W. (2016). Phys. Chem. Chem. Phys..

[cit58] Flores N. M., Pal U., Galeazzi R., Sandoval A. (2014). RSC Adv..

[cit59] Tse G. (2021). Computational Condensed Matter.

[cit60] Liu S., Li C., Yu J., Xiang Q. (2011). CrystEngComm.

[cit61] Qiao F., Liang Q., Hou X., Yang J., Xu Q. (2019). J. Electron. Mater..

